# The effect of integrative neuromuscular training on physical fitness and punching performance in highly-trained male boxers

**DOI:** 10.3389/fphys.2026.1810393

**Published:** 2026-05-12

**Authors:** Lei Zhang, Hanyi Li, Qiang Wei, Youlin Xiao, Qin Gao

**Affiliations:** 1 The General Education College of Wenzhou Business College, Wenzhou, Zhejiang, China; 2 College of Physical Education, Dalian University, Dalian, Liaoning, China

**Keywords:** athletic performance, continuous punch, periodized training, physical fitness training, punching power

## Abstract

**Purpose:**

This study investigates the effects of a 3-week Integrative Neuromuscular Training (INT) on the physical fitness and punching ability of male boxers preparing for international competitions.

**Methods:**

Eighteen male amateur boxers from the Chinese national team (age: 22.50 ± 1.98 years; height: 182.22 ± 8.49 cm; weight: 75.97 ± 14.19 kg; training experience: 8.44 ± 1.61 years), categorized into lightweight, middleweight, and heavyweight (six athletes per category), completed a 3-week INT program including resistance strength, plyometric, core stability, functional, speed, and coordination/agility training.

**Results:**

In the physical fitness testing, significant improvements were observed in the bench press (P<0.001, ES = 1.13), squat (P<0.001, ES = 1.72), countermovement jump testing (P<0.001, ES = 1.14), 400-m sprint (P<0.001, ES = 1.26), 1-minute hexagon jump (P<0.001, ES = 1.73), and 3-minute double-under testing (P = 0.001, ES = 1.73). No significant differences were found in the 30-m sprint (P = 0.593, ES = 0.13) and 3000-m run test (P = 0.144, ES = 0.36); in the punching performance testing, changes were considerable in the cross (P = 0.001, ES = 0.95), lead hook (P<0.001, ES = 1.26), lead uppercut (P = 0.003, ES = 0.80), jab (P = 0.009, ES = 0.70), and rear hook (P<0.001, ES = 1.98). The rear uppercut (P = 0.096, ES = 0.42) did not show a significant difference. Additionally, significant enhancements were observed in the 10-s cumulative punching power (P = 0.001, ES = 0.98), 30-s cumulative punching power (P = 0.009, ES = 0.70), and 3-minute cumulative punching power (P<0.001, ES = 1.12).

**Conclusion:**

Short-term INT can effectively enhance the athletic performance of male boxers. An INT program implemented within the preparatory period is beneficial toward both fitness and punching ability, thus facilitating boxers to move more easily into high-load training.

## 1 Introduction

Integrative neuromuscular training (INT) is a multicomponent training approach that combines functional movement exercises with strength, power, coordination, speed, agility, balance/stability, and plyometric exercises within a structured program (Fort-Vanmeerhaeghe et al., 2016; Myer et al., 2011). Previous studies have shown that INT can improve selected aspects of physical fitness and athletic performance and may also help reduce sports injury risk in both general and athletic populations (Akbar et al., 2022; Fort-Vanmeerhaeghe et al., 2016; Myer et al., 2011). Reported benefits of INT include improvements in muscular strength, balance, and vertical jump performance, although the specific outcomes vary according to the training content, intervention duration, and target population (Canli, 2019; Dhawale et al., 2020; Filipa et al., 2010; Gavala et al., 2023; Lin et al., 2022; Nunes et al., 2021). Therefore, INT may be a useful training strategy when multiple performance-related physical qualities need to be developed in an integrated manner.

Boxing is a combat sport that places high demands on strength, power, speed, endurance, agility, coordination, and sport-specific punching ability (Chaabene et al., 2015; Dinu et al., 2020). Elite boxers must repeatedly execute explosive offensive and defensive actions throughout the bout while maintaining effective technical and tactical performance (Davis et al., 2015; El Ashker, 2011). Because boxing performance depends on the coordinated contribution of multiple physical qualities, preparatory-phase conditioning should not only increase general fitness but also support the physical capacities underpinning punching performance (Chaabene et al., 2015; Loturco et al., 2016). In this context, INT is not intended to replace traditional strength and conditioning in boxing, but rather to integrate multiple physical-training components within a coordinated weekly structure. In the present study, this approach included resistance strength, plyometric, core stability, functional, speed, and coordination/agility training, with an emphasis on enhancing explosive power, strength, speed, and functionality while addressing the specific physical demands of boxing.

From a periodization perspective, physical conditioning is a continuous aspect of a boxer’s career, with fitness levels fluctuating according to the frequency of competitions. A well-structured training plan can help boxers achieve appropriate physical readiness at different stages of the season (Issurin, 2010). During the preparatory phase, athletes are expected to establish a broad physical foundation through aerobic and anaerobic training while also developing muscular endurance and strength reserves to support subsequent technical training and competition (Franchini et al., 2015). Because this phase is typically characterized by high training volume and intensity but relatively lower competitive pressure, it provides an appropriate context for comprehensive physical development. In this regard, INT may be particularly suitable during the preparatory phase because it integrates multiple training components within a coordinated structure and may help address several physical demands simultaneously (Fort-Vanmeerhaeghe et al., 2016; Myer et al., 2011). Therefore, incorporating INT into the preparatory stage of a major competition cycle may be beneficial for elite boxers.

Recent INT-related research has expanded beyond youth populations and has increasingly been applied in trained and elite athletes (Akbar et al., 2022). However, evidence in boxing remains limited. Existing boxing studies have mainly focused on physical and physiological characteristics, as well as the relationship between strength and power qualities and punching performance (Chaabene et al., 2015; Loturco et al., 2016), whereas studies examining a structured multicomponent INT program and its simultaneous effects on both physical fitness and punching performance in boxers are still scarce. A recent study reported that a 3-week INT intervention improved athletic-performance-related outcomes in elite female boxers (Niu et al., 2024), but evidence in elite male boxers remains limited. Based on the characteristics of the present program, we hypothesized that resistance strength and plyometric training would primarily contribute to punch force production, core stability and functional training would support force transfer through the kinetic chain, and speed as well as coordination/agility training would contribute to punch execution and repeated punching performance. Therefore, this study aimed to examine the short-term effects of a 3-week INT intervention on the physical fitness and punching performance of elite male boxers during the preparatory phase.

## 2 Methods

### 2.1 Participants

Eighteen elite amateur male boxers from the Chinese national team were recruited for the study (age 22.50 ± 1.98 years; height 182.22 ± 8.49 cm; weight 75.97 ± 14.19 kg; training experience 8.44 ± 1.61 years, all with experience in major AIBA competitions). The participants were evenly divided into three weight categories: lightweight, middleweight, and heavyweight, with six boxers in each category. Among them, eight were left-handed, and the dominant hand was defined as the rear hand in the punching ability test. The boxers implemented the INT plan during the physical conditioning phase of their winter training camp in January 2022, in preparation for the International Men’s Boxing Championships in Tashkent in April 2023. All participants had no injuries for two months prior to the testing and successfully completed the three-week INT program. This study was approved by the Research Ethics Committee of Shanghai University of Sport (No. 102772021RT102) and conducted in accordance with the principles of the Declaration of Helsinki. Written informed consent was obtained from all participants, with each individual signing the informed consent form prior to study participation.

### 2.2 Procedures

#### 2.2.1 Experimental protocols

**Figure 1:**
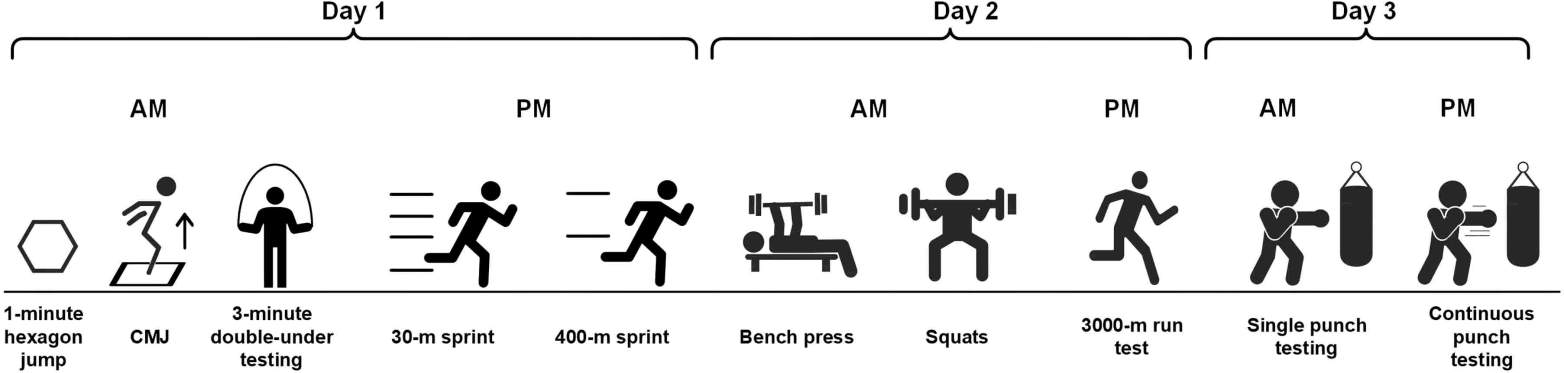
Testing process. The pre- and post-intervention assessments were conducted over 3 consecutive days. On Day 1, athletes completed the 1-minute hexagon jump, countermovement jump (CMJ), 3-minute double-under, 30-m sprint, and 400-m sprint tests. On Day 2, bench press, squat, and 3000-m run tests were performed. On Day 3, single-punch testing and continuous-punch testing were conducted. AM, morning session; PM, afternoon session.

This study used a single-group pre–post intervention design. The experimental procedure consisted of a pre-intervention assessment, a 3-week INT intervention, and a post-intervention assessment. Both the pre-test and post-test were completed over the same 3-day schedule and in the same testing order shown in **Figure 1** to minimize potential order effects. The general testing schedule and INT framework were informed by a recent 3-week INT study in elite female boxers (Niu et al., 2024), but the present study was implemented in elite male boxers during the preparatory phase. All athletes were familiar with the general physical-fitness and punching-performance tests through routine national-team training and monitoring practices before the study. Before each testing day, athletes performed a standardized warm-up, and subsequent tests were conducted only after routine physiological recovery.

The INT intervention lasted 3 weeks and included six components: resistance strength training, plyometric training, core stability training, functional training, speed training, and coordination/agility training (**Table 1**). Training was organized across six training days per week (Monday to Saturday) using morning and afternoon sessions, with Sunday scheduled as a rest day. According to the weekly plan, some components were performed as separate sessions, whereas others were combined within the same day. The training contents for each component were adapted from previous studies (Fort-Vanmeerhaeghe et al., 2016), tailored to the physical development of the athletes and the specific demands of boxing, with an emphasis on enhancing explosive power, strength, speed, and functionality. Each component included 6–8 exercises, and the specific loads and set structures are shown in **Table 2**. Rest intervals were standardized according to training type: 3 min between sets for resistance strength training and 2 min between sets for plyometric, speed, and coordination/agility training, in order to facilitate recovery before the next effort. Each session lasted approximately 2 h, including around 20 min of warm-up and 20 min of stretching activities.

**Table 1:** Overview of the 3-week daily type of training.

Training Content								
Week	Time	Monday	Tuesday	Wednesday	Thursday	Friday	Saturday	Sunday
W1								
	AM	Coordination and Agility Training	Resistance Strength Training	Core Control Training,	Coordination and Agility Training	Core Control Training	Resistance Strength Training	Off
	PM	Functional Training	Plyometrics Training,	Off	Core Control Training,	Speed Training,	Functional Training	Off
			Aerobic training		Aerobic training	Aerobic training		
W2								
	AM	Core Control Training	Resistance Strength Training	Speed Training,	Coordination and Agility Training	Resistance Strength training	Speed Training	Off
				Aerobic training				
	PM	Speed Training	Plyometrics Training,	Off	Plyometrics Training	Coordination and Agility Training	Functional Training	Off
			Aerobic training		Aerobic training			
W3								
	AM	Resistance Strength Training	Plyometrics Training	Plyometrics Training	Core Control Training	Plyometrics Training	Core Control Training	Off
	PM	Speed Training	Plyometrics Training,	Speed Training	Aerobic training	Coordination and Agility Training	Functional Training	Off
			Aerobic training					

W1-3, training weeks; AM, morning training; PM, afternoon training.

**Table 2:** INT content arrangement.

Training component	Training content	Training Load	RM or Time	Sets
Resistance Strength Training	Bench press	3RM	5 reps	3
	Squat	3RM	5 reps	3
	Seal row bench	3RM	5 reps	3
	Deadlift	3RM	5 reps	3
	Barbell hip thrust	3RM	5 reps	3
	Power clean	5RM	8 reps	3
	Weighted calf raise	20kg	15 reps	3
Plyometrics Training	Box jump	85% maximum vertical jump height	6 reps	4
	Rotational box jump	85% maximum vertical jump height	6 reps	4
	Vertical jump with elastic band	Body weight	6 reps	4
	Alternating lunge jump	Body weight	6 reps × 2	4
	Alternating kettlebell squat and press	15kg	20 reps × 2	3
	Downward smash with battle ropes	10kg	20 reps	3
	Medicine ball overhead throw	5kg	15 reps	2
	Medicine ball wall slam	5kg	15 reps	2
Core Control Training	Plank	Body weight	2 min	3
	Standing resistance band anti-rotation training	Body weight	2 min	3
	Eight-level plank	Body weight	30s + 20s × 6 + 60s	2
	Crocodile walk	Body weight	20m×2	2
	Bear walk	Body weight	20m×2	2
	TRX plank	Body weight	60 reps	2
	TRX knees in crunch	Body weight	60 reps	2
	TRX Jackknife	Body weight	60 reps	2
Functional Training	Barbell overhead press and slam	15kg or 10kg	30 reps	2
	Barbell around the shoulders	15kg or 10kg	30 reps	2
	Barbell kneeling press	15kg or 10kg	30 reps	2
	Kettlebell deadlift	15kg or 10kg	30 reps	2
	Kettlebell swing	15kg or 10kg	30 reps	2
	Cannonball lateral movement	15kg or 10kg	2 min	2
	Battle rope training	Body weight	2 min	2
	Climbing machine	Body weight	5 min	2
Speed Training	Short-distance sprint	20m	1 reps	2
	Shuttle sprint	6 × 5m	1 reps	4
	High knees sprint	20m	1 reps	4
	Low-intensity resistance running	50m	1 reps	3
	Moderate-intensity resistance running	50m	1 reps	3
	Hill running	10m	1 reps	3
Coordination and Agility Training	Hexagon jump	Body weight	120 reps	3
	Rope ladder lateral movement	5m	1 reps	3
	Rope ladder diagonal step	5m	1 reps	3
	Hurdle drills	Hurdle height 25cm	12 reps	3
	Lateral skater hops	10m	1 reps	3
	Dot drills	Body weight	120 reps	3
	Shape footwork training	Body weight	10 reps	3
	Shadowboxing	Body weight	2 min	3

RM, Repetition maximum; reps, repetitions; TRX, Total Resistance Exercise.

In addition, aerobic training was incorporated into the weekly schedule according to the winter-training plan, and each aerobic training session consisted of two 3000-m runs. The athletes remained in the national-team winter-training environment during the intervention period and continued their routine boxing-specific training according to the camp schedule, whereas the INT program served as the structured physical-conditioning component of that schedule. Post-intervention testing was conducted under the same standardized conditions as the pre-test to reduce the likelihood that acute cumulative fatigue affected test performance.

#### 2.2.2 Assessment of physical fitness variables and punching performance

Before each testing session, participants completed a standardized warm-up consisting of general aerobic activity, dynamic mobility exercises, and task-specific submaximal rehearsal trials.

*Maximum strength testing* Upper-body and lower-body maximal strength were assessed using the bench press and squat, respectively. Participants progressively increased the load from light to heavy following specific warm-up sets. The heaviest load successfully completed with proper technique for 3, 4, or 5 repetitions was recorded, with a maximum of five attempts allowed and 3 min rest between attempts. The exact number of completed repetitions for each participant was then entered into Brzycki’s formula (Brzycki, 1993): 1RM = 100 × weight lifted/(102.78 – 2.78 × number of repetitions), and the estimated 1RM was used as the maximal strength value.

*30-m sprint* Speed for boxers was measured using a light gate system (Smartspeed, Fusion Sport Inc., Australia). The participants adopted a standing start position, initiated the sprint at their own discretion after preparation, passed through the first light gate, and concluded the test after passing through the second light gate. Two testing attempts were given, and the best result was recorded, with precision up to 0.01 seconds.

*Countermovement jump testing (CMJ)* Lower-limb explosive power was assessed using a vertical jump mat (Smartjump, Fusion Sport Inc., Australia). Participants stood upright on the mat, performed a rapid countermovement, and jumped vertically with maximal effort. They were instructed to land safely in place with natural flexion of the hips and knees. Three trials were performed, and the best result was recorded to the nearest 0.01 cm.

*400-m sprint* Anaerobic running performance was assessed using a 400-m sprint. Participants started from a standing position, and time was recorded when the torso crossed the finish line, to the nearest second.

*3000-m run test* Aerobic endurance was assessed using a 3000-m run test. Participants started from a standing position, and time was recorded when the torso crossed the finish line, to the nearest second.

*Coordination and agility testing* Coordination and agility were assessed using the 1-minute hexagon jump and the 3-minute double-under test. In the 1-minute hexagon jump, participants stood with both feet inside the hexagon and performed two-foot jumps outward and back in a clockwise pattern; the total number of successful jumps completed within 1 min was recorded. In the 3-minute double-under test, participants attempted to complete as many correctly executed double-unders as possible within 3 min. Missed attempts were not counted, but participants were allowed to continue until the end of the test period. The total number of successful double-unders was recorded.

*Punching performance testing* Punching performance was assessed using boxing testing equipment (Xingxun, Shanghai, China) for single-punch and continuous-punch tests. The device incorporates 3D accelerometers within the boxing gloves to record punch motion. Before each testing session, the system was initialized and checked according to the manufacturer’s instructions. Punching power outputs were generated by the device software from accelerometer-derived motion signals using the manufacturer’s built-in algorithm. Detailed information on sensor sampling frequency and the proprietary processing algorithm was not available from the manufacturer to the investigators. The use of accelerometer-based punch monitoring in boxing has been reported in previous studies (Lambert et al., 2018; López-Laval et al., 2020; Omcirk et al., 2021). In the single-punch test, subjects adopted a free-standing position according to their dominant side and prepared for punching. They performed five straight punches, five hooks, and five uppercuts with the dominant and non-dominant hands in sequence, exerting maximal effort in each trial. The best value for each punch type was recorded. In the continuous-punch test, subjects again prepared for punching according to their dominant side. Upon command, they initiated the testing system and performed continuous punching with maximal effort for 10 s, 30 s, and 3 min, respectively, and cumulative punching power was calculated at the end of each test. During the tests, the boxers adjusted punching distance and timing in response to the target, thereby maintaining a more dynamic interaction with the device and better simulating competitive punching conditions.

### 2.3 Statistical analyses

The data were statistically analyzed using IBM SPSS 27.0 software and are presented as mean ± standard deviation (SD). Normality of each dataset was assessed using the Shapiro-Wilk test. Paired-sample t-tests were conducted on pre- and post-intervention results after confirming normal distribution. A significance level of P < 0.05 was considered indicative of statistical significance. Effect size (Cohen’s d) was calculated to evaluate the magnitude of change in pre- and post-intervention measurements, with effect sizes categorized as small (0.2 < ES < 0.5), medium (0.5 < ES < 0.8), and large (ES > 0.8) based on established criteria (Rhea, 2004).

## 3 Results

The results of the pre- and post-intervention tests are presented in **Table 3**.

**Table 3:** Comparison of testing protocols before and after 3-week INT.

	Indicator	Testing protocols	Pre	Post	P	ES
Physical Fitness	Strength	Bench press (kg)	93.50 ± 16.32	99.22 ± 17.89	<0.001	1.13
		Squat (kg)	116.39 ± 13.28	136.11 ± 17.87	<0.001	1.72
	Explosive power	CMJ (cm)	40.17 ± 4.08	43.28 ± 4.70	<0.001	1.14
	Speed	30-m sprint (s)	4.45 ± 0.25	4.47 ± 0.21	0.593	0.13
	Endurance	400-m sprint (s)	60.61 ± 1.65	59.33 ± 1.91	<0.001	1.26
		3000-m run test (s)	647.72 ± 41.81	653.33 ± 40.57	0.144	0.36
	Coordination and Agility	1-minute hexagon jump (pcs)	61.83 ± 6.54	75.28 ± 5.95	<0.001	1.73
		3-minute double-under (pcs)	273.94 ± 58.46	293.94 ± 45.95	0.001	0.93
Punching Performance	Single punch (Non-dominant hand)	Jab (kw)	20.63 ± 4.18	27.95 ± 8.47	0.001	0.95
		Hook (kw)	24.69 ± 7.46	38.82 ± 10.41	<0.001	1.26
		Uppercut (kw)	21.49 ± 4.54	27.97 ± 8.72	0.003	0.80
	Single punch (Dominant hand)	Cross (kw)	25.24 ± 6.84	31.81 ± 12.36	0.009	0.70
		Hook (kw)	32.78 ± 8.18	50.61 ± 13.85	<0.001	1.98
		Uppercut (kw)	30.75 ± 10.27	38.23 ± 18.25	0.096	0.42
	Special explosive power	10-s cumulative punching power (kw)	892.33 ± 210.53	1170.95 ± 190.02	0.001	0.98
	Special endurance	30-s cumulative punching power (kw)	2195.46 ± 415.65	2547.63 ± 376.50	0.009	0.70
		3-minute cumulative punching power (kw)	6892.01 ± 1481.75	8313.83 ± 1926.21	<0.001	1.12

CMJ, Countermovement Jump; Pre, testing before 3-week INT; Post, testing after 3-week INT; ES, effect size.

### 3.1 Changes in physical fitness variables

**Figure 2:**
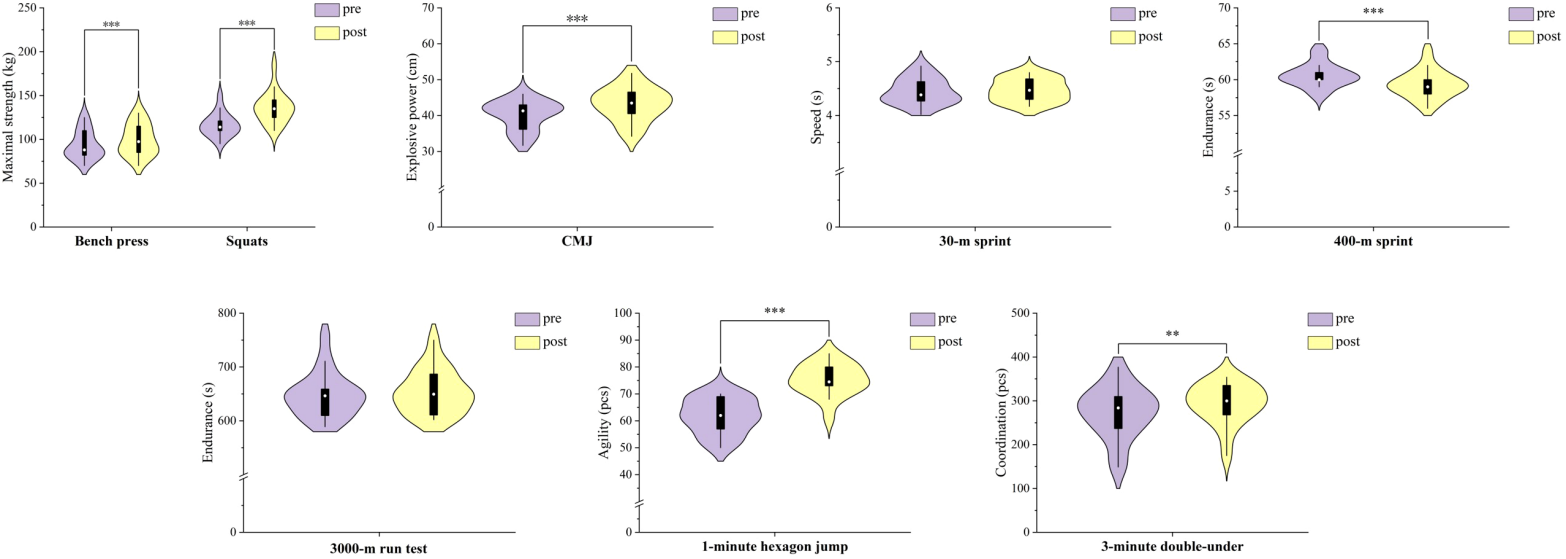
Effect of INT on physical fitness. Changes in physical fitness variables before (pre) and after (post) the 3-week integrative neuromuscular training (INT) intervention in elite male boxers. The figure presents results for bench press, squats, countermovement jump (CMJ), 30-m sprint, 400-m sprint, 3000-m run test, 1-minute hexagon jump, and 3-minute double-under. Data are shown as violin plots with embedded boxplots. **P < 0.01 and ***P < 0.001 versus pre.

Significant improvements were observed in bench press (P < 0.001, ES = 1.13), squat (P < 0.001, ES = 1.72), CMJ (P < 0.001, ES = 1.14), 400-m sprint (P < 0.001, ES = 1.26), 1-minute hexagon jump (P < 0.001, ES = 1.73), and 3-minute double-under (P = 0.001, ES = 0.93). No significant differences were found in the 30-m sprint (P = 0.593, ES = 0.13) or the 3000-m run test (P = 0.144, ES = 0.36). These changes are illustrated in **Figure 2**.

### 3.2 Changes in single-punch performance

**Figure 3:**
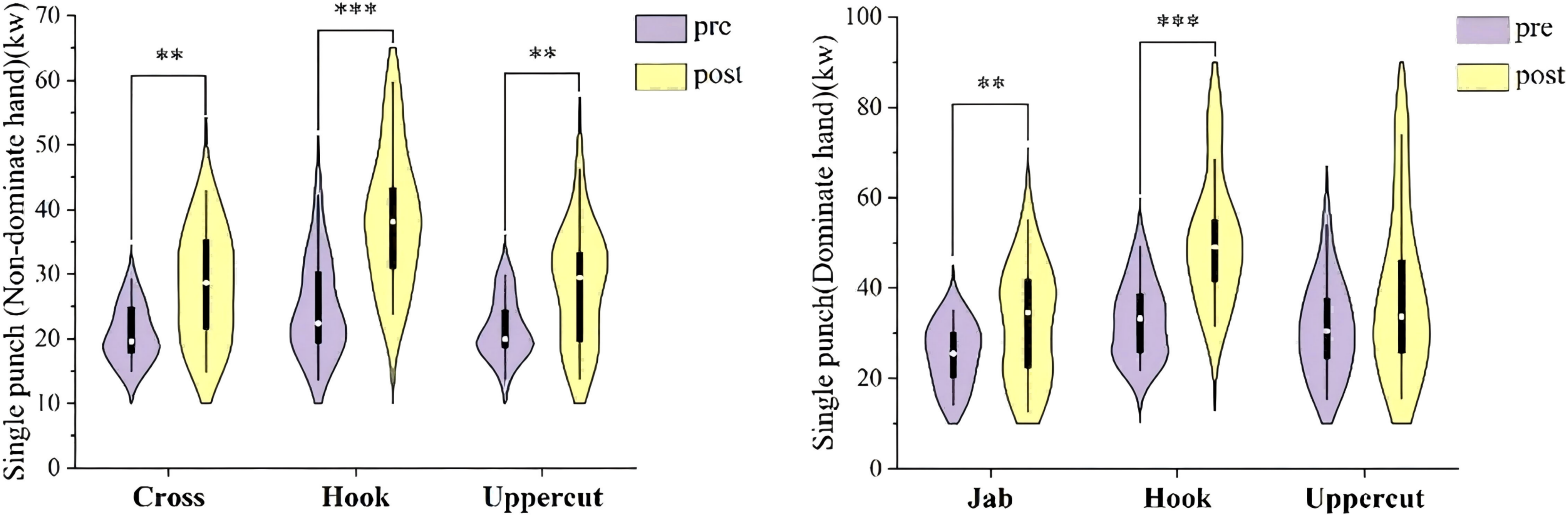
Effect of INT on single punch power. Changes in single-punch power before (pre) and after (post) the 3-week integrative neuromuscular training (INT) intervention in elite male boxers. The left panel shows non-dominant-hand punching performance (cross, hook, and uppercut), and the right panel shows dominant-hand punching performance (jab, hook, and uppercut). Data are shown as violin plots with embedded boxplots. **P < 0.01 and ***P < 0.001 versus pre.

Significant improvements were found in the cross (P = 0.001, ES = 0.95), lead hook (P < 0.001, ES = 1.26), lead uppercut (P = 0.003, ES = 0.80), jab (P = 0.009, ES = 0.70), and rear hook (P < 0.001, ES = 1.98). The rear uppercut did not show a significant difference (P = 0.096, ES = 0.42). These changes are illustrated in **Figure 3**.

### 3.3 Changes in cumulative punching performance

**Figure 4:**
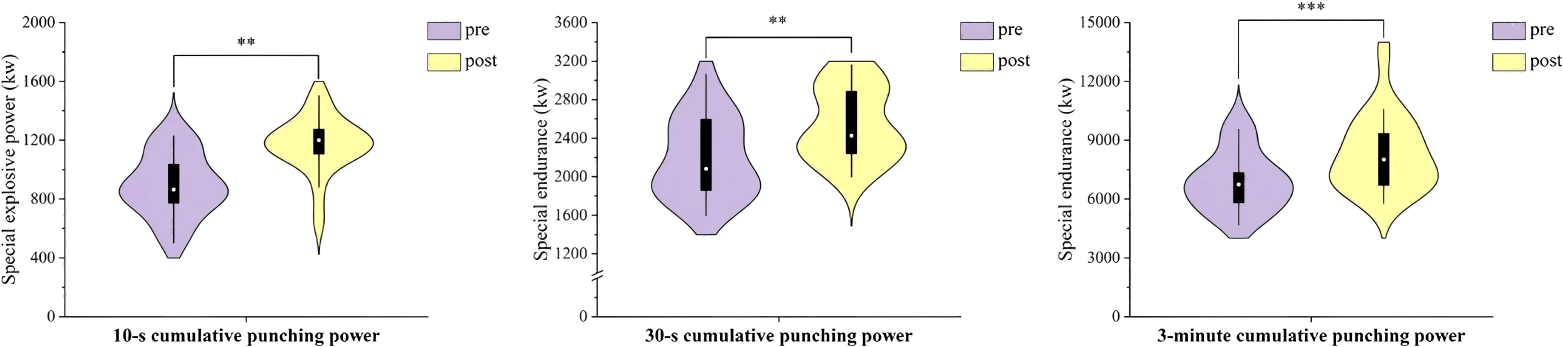
Effect of INT on cumulative punching power. Changes in cumulative punching power before (pre) and after (post) the 3-week integrative neuromuscular training (INT) intervention in elite male boxers. The figure shows cumulative punching power over 10 s, 30 s, and 3 min. Data are presented as violin plots with embedded boxplots. **P < 0.01 and ***P < 0.001 for pre-post comparisons.

Significant improvements were also observed in 10-s cumulative punching power (P = 0.001, ES = 0.98), 30-s cumulative punching power (P = 0.009, ES = 0.70), and 3-minute cumulative punching power (P < 0.001, ES = 1.12). These changes are illustrated in **Figure 4**.

## 4 Discussion

Physical fitness training is an essential component of boxing and provides an important foundation for the execution of boxing techniques. In the present single-group pre–post study, improvements were observed in several physical-fitness variables after the 3-week INT program. These findings suggest that such a program may be useful during the preparatory phase in highly trained boxers. However, given the absence of a control or comparison group, the observed changes should be interpreted cautiously and should not be attributed exclusively to INT.

### 4.1 Impact of INT on boxers’ physical fitness

In the maximum strength testing, the bench press mimics the punching motion, particularly the straight punch technique (López-Laval et al., 2020), while the squat is a reliable indicator of lower body strength in boxers (Loturco et al., 2016). After the 3-week INT, significant improvements were observed in both bench press and squat (**Figure 2**), with a more pronounced increase in lower body strength (ES = 1.72) compared to upper body strength (ES = 1.13). Strength is an important physical component of boxing performance, although boxing performance also depends on technical, tactical, and perceptual factors in addition to physical capacity (Beattie and Ruddock, 2022; Bingül et al., 2018). The present improvements in maximal strength may be related to the resistance-strength component of the INT program. Additionally, strength is important for injury prevention (Lloyd et al., 2015), indicating that resistance-oriented INT may be helpful for restoring or enhancing strength reserves in highly trained boxers. It is important to design resistance training loads in the INT program to target muscles at maximal or near-maximal rates (Cronin and Sleivert, 2005).

Loturco et al. (2016) found significant correlations between the rear hand straight punch (r=0.72) and the lead hand straight punch (r=0.80) with CMJ height in elite Olympic boxers, establishing CMJ as an indicator of explosive power in boxers. The INT program significantly improved the explosive power of the boxers (ES = 1.14) (**Figure 2**), consistent with findings in volleyball (Gavala et al., 2023; Nunes et al., 2021), soccer (Filipa et al., 2010), and other sports. The present intervention included plyometric, coordination/agility, and resistance-strength training, all of which may have contributed to the observed improvement in CMJ performance. One study found that an 8-week INT program did not improve and even decreased CMJ in male skiers (Vitale et al., 2018).

In **Figure 2**, the 30-meter sprint test results showed no significant changes between the pre-test and the post-test performances. Hydar Abbas et al. (2019) and Canli (2019) suggest that training at an intensive training (INT) can improve abilities related to speed. For example, Canli’s 12-week INT program led to a small improvement in the 20-meter sprint time of 12 male basketball players, reducing their time from 4.1 seconds to 4.0 seconds. However, other studies, like Lindblom et al. (2012), did not find any improvement in sprinting ability among adolescent female soccer players after a 9–10 week INT program. Similarly, Mendiguchia et al. (2015) reported that only minimal improvements in sprint performance for 27 amateur male soccer players with an effect size of 0.32 for the 5-meter sprint and a negative effect size of -0.13 for the 20-meter sprint after 7 weeks of INT. Some researchers such as Brooks et al. (2013) have argued that INT might be more effective in improving explosive power and movement control in female athletes compared to males. In addition, studies show that training programs lasting at least 6 weeks with sessions lasting no less than 90 minutes tend to be more effective for enhancing speed-related qualities. Given the relatively short intervention period and the high fitness levels of the male boxers in this study, their baseline speed was already quite advanced, making it difficult to achieve further improvements. While plyometric training did lead to improvements in lower body explosive power, these gains were not sufficient to significantly enhance the boxers’ sprinting speed. Overall, the effect of INT on speed development remains unclear and requires further investigation across different sports disciplines.

Significant differences were found in the 400-meter run test, but no such changes were observed in the 3000-meter run (**Figure 2**). Amateur boxing has become increasingly intense and dynamic due to evolving rules (Chaabene et al., 2015). Elite Olympic boxers are required to maintain about 1.4 actions per second during a match, including approximately 20 punches, 2.5 defensive moves, and 47 vertical hip movements per minute, across three 200-second rounds (Davis et al., 2015). Boxers must maximize their punch output and avoid opponent punches within the limited match time (3x3 minutes), with only a one-minute break between rounds. As a result, boxing training places a strong emphasis on anaerobic capacity, while also maintaining a high level of aerobic capacity (Chaabene et al., 2015), since anaerobic capacity is crucial during critical moments of a fight. However, there is no research has been explored the effects of INT on both anaerobic and aerobic endurance levels. The improvement in anaerobic endurance was found in this study may be attributed to adaptations from resistance strength training and speed training (Berryman et al., 2018; Moore et al., 2019). This improvement likely stems not from an increased lactate threshold, but from better running economy (Skovgaard et al., 2014) and enhanced lower limb power output (Beattie et al., 2017). Speed training also involves short sprints and intervals. It can improve skeletal muscle oxidative capacity and respiratory regulation and leading to better anaerobic endurance (Freese et al., 2013). The variation in aerobic capacity among elite male boxers during regular training cycles may be relatively small, even though aerobic training was included in the present study. The lack of improvement in aerobic endurance may be related to the short intervention period as significant progress in aerobic endurance generally requires longer and more focused training (Wilson et al., 2012). It is also worth noting that there can be an interaction effect between resistance strength training and aerobic endurance improvements (Wilson et al., 2012). Both anaerobic and aerobic capacities are important in boxing. In particular, the aerobic energy system contributes to recovery between exchanges and rounds and helps maintain work rate throughout the bout. Therefore, the non-significant 3000-m result in the present study should not be interpreted as evidence that aerobic fitness is unimportant in boxing.

In boxing, coordination and agility are important composite physical qualities. The 1-minute hexagon jump test has been identified as a reliable measure of agility (Beekhuizen et al., 2009) and is used in broader athletic agility evaluations (Hernández-Davó et al., 2021). The 3-minute double-under test is also relevant to boxing because it requires rhythm, repeated lower-limb actions, and coordination over a duration similar to that of one boxing round (Chottidao et al., 2022; Miyaguchi et al., 2015). Previous studies indicate that neuromuscular-oriented training can improve coordination- and agility-related performance in athletes (Dhawale et al., 2020; Filipa et al., 2010; Steffen et al., 2013). In the present study, improvements were observed in the 1-minute hexagon jump test (ES = 1.73) and the 3-minute double-under test (ES = 0.93) after 3 weeks of INT. These changes may reflect the contribution of coordination/agility, plyometric, and resistance-strength training. However, because proprioceptive and central nervous system adaptations were not directly assessed in the present study, these possible explanations should be interpreted cautiously rather than as direct findings.

### 4.2 Impact of INT on boxers’ punching performance

Current research on the key factors influencing boxing performance is relatively scarce, with many studies concentrating on movement kinetics (Stojsih et al., 2010), punching performance (Loturco et al., 2018; Turner et al., 2011), and strength attributes (Chaabene et al., 2015; Lenetsky et al., 2013). These studies underscore the widely acknowledged significance of punch effectiveness in boxing (Lenetsky et al., 2013; Loturco et al., 2021). Punching necessitates swift, multi-planar muscular actions, propelling the fist towards the opponent’s body or head (Dinu et al., 2020). A greater punch impact force provides boxers with an advantage, boosting the likelihood of landing effective strikes, wearing down opponents and potentially leading to knockouts or knockdowns (Bingül et al., 2018; Dunn et al., 2019; Liu et al., 2022). When comparing pre- and post-INT intervention test results, there were notable enhancements in the power of various punches, including the lead hand and rear hand straight punches, hooks, and uppercuts (cross: ES = 0.95, lead hook: ES = 1.26, lead uppercut: ES = 0.80; jab: ES = 0.70, rear hook: ES = 1.98). Although the rear uppercut’s improvement was not statistically significant (P = 0.096, ES = 0.42), it still showed an upward trend (**Figure 3**). To date, there are no studies dedicated solely to punching power in boxing. Punching power can be defined as the energy or force delivered per unit time while serving as a crucial measure of a boxer’s explosiveness and technical proficiency. Enhancing both punch speed and strength can elevate punching power and improving competitive performance. Among various punch types, hooks demonstrated the highest impact force compared to straight punches and uppercuts (Dunn et al., 2019; Smith, 2006). Dinu et al. (2020) further found that hooks possess the highest punching velocity followed by straight punches while making hooks the most powerful and offering the greatest potential for improvement. This study’s finding, showing that INT significantly improved the power of both lead and rear hand punches, especially hooks, aligns with previous research. Numerous studies reveal that the neuromuscular system’s ability to generate force is a critical factor limiting punch performance (Dunn et al., 2022; Loturco et al., 2016). Findings present that the inclusion of neuromuscular training in boxing training programs can immensely augment force production and punch power. It also supports the idea that neuromuscular training enhances a boxer’s physical ability by increasing muscle activation and neural coordination. In fact, according to Niu et al. (2024), neuromuscular protocols can improve punching power, with individuals using it not only for punching but also to improve muscle coordination for punching power. INT seeks to enhance these variables as a means to improve athletic performance, such as central nervous system sensitivity and control, motor units, and muscle activation (Eliason et al., 2023). Another research conducted by Niu et al. (2024) on the INT program shows that the strength training part of it leads to great increases in maximal strength of upper and lower limbs, which are the key to punch power generation. However, considerable improvements in the maximal strength of both the upper and the lower limbs were observed, which resulted from the primary strength training component in the INT program. A previous study found that there was a very strong correlation (r = 0.7) between a boxer’s maximal punch velocity and the bench press strength (Loturco et al., 2016). The lower body strength also plays a central role in boxing performance (Chaabene et al., 2015; Lenetsky et al., 2013). Boxers generate force through their lower limbs and transferring this force via hip and torso rotation to deliver the punch during a punch (Zhou et al., 2022). Awana et al. (2023) emphasized that activating both upper and lower body muscle groups together during a punch enables more efficient force transfer while improving the power of different punch types, especially the hook which benefits from such synergy. Plyometric training enhanced the boxers’ lower body explosive power while core control training improved their core strength. It also promotes the efficient transfer of force which from the lower body then to the upper body and ultimately to the fist. These training components were especially profitable in increasing punch velocity and impact force. Awana et al. (2023) suggested that plyometric exercises may facilitate rapid muscle fiber recruitment during explosive movements commonly required in boxing. In the present study, the observed increase in punch power may be associated with improvements in strength- and explosiveness-related physical qualities. Coordination and agility may also have contributed to punching performance, although their relative contribution was not directly examined in the present study.

The chances of winning with a single decisive blow when the other makes a mistake are quite low in boxing matches where both opponents have similar skill levels. For this reason, boxers must stay composed and manage their stamina and rhythm throughout long matches. The ability to throw consecutive punches is a clear demonstration of a boxer’s overall physical strength. Among the indicators used to measure consecutive punching ability, the 10-second cumulative punching power is a key metric to evaluate a boxer’s explosive power. During a match, when a boxer finds an advantageous opening to attack, they need to deliver a series of punches in a very short time (less than 10 seconds) to knock down their opponent. On the other hand, the 30-second and 3-minute cumulative punching power are more conventional indicators to assess a boxer’s anaerobic endurance. The 30-second cumulative punching power mirrors the glycolytic capacity and showing the level of the wellness boxers can perform under high-intensity conditions and manage energy over a short duration. Meanwhile, the 3-minute cumulative punching power aligns with the length of each round in a boxing match and testing the boxer’s ability to maintain punching effectiveness under the extreme stress of completing a full round game. The INT intervention led to significant improvements in the boxers’ 10-second, 30-second, and 3-minute cumulative punching power (**Figure 4**). Among these, the 3-minute cumulative punching power showed the greatest improvement (ES = 1.12), while the 30-second cumulative punching power demonstrated the smallest enhancement (ES = 0.70). Results indicate that the boxers have improved their ability to sustain attacks over short bursts and endure the intensity of entire rounds. One possible explanation for the improvement in continuous punching performance is the concurrent increase in single-punch power, which may have helped the boxers deliver more forceful punches during repeated punching efforts. Coordination and agility play a highly crucial role in the consecutive punch tests when compared to single punch tests. Boxing requires precise striking and dodging skills which demand the ability to quickly change body position and movement direction during attacks or defense (Zhong and Bu, 2022). Strong body coordination supports the execution of complex movements and helps conserve energy (Davis et al., 2013) and enabling boxers to react more effectively to shifts in the position of their opponent or the punching bag while make accurate decisions and adjust their stance for more fluid and powerful punching. Additionally, these improvements in anaerobic endurance also contribute significantly to perform better in the 30-second and 3-minute cumulative punch tests. Given the intensity of amateur boxing matches (Chaabene et al., 2015), fatigue is a major factor that affects performance (El Ashker, 2011). To enhance the efficiency of the anaerobic energy system enables boxers to sustain high levels of punching power and maintain a dominant state during matches.

In conclusion, the physical fitness level of boxers is directly related to their athletic performance. High-level boxers should ensure they have no significant physical shortcomings. Although the INT program in this study did not include specific boxing training content (e.g., punching bags, sparring), it was designed based on movements, force patterns, and energy metabolism principles closely related to boxing. Emphasizing the conversion between physical fitness and specific skills, the program not only enhanced basic physical fitness but also positively impacted the single and continuous punch abilities of male boxers.

### 4.3 Limitation and future outlook

This study has several limitations and shortcomings: (1) The lack of a control group means that it is not possible to directly compare the effects of short-term INT intervention with other training methods for boxers, and it also limits causal inference. Although improvements were observed after the 3-week INT program, it cannot be determined to what extent these changes were specifically attributable to INT rather than to concurrent training, normal variation during the training camp, or test familiarity; (2) All participants in this study were male boxers, which limits the generalizability of the findings to female boxers and other combat-sport populations; (3) The intervention period lasted only 3 weeks, so this study could only examine the short-term effects of INT in boxers. While this duration may be sufficient to detect short-term changes in some performance variables, it may be insufficient to capture longer-term adaptations, especially in highly trained athletes whose performance capacities are already relatively stable. Future studies should consider using control or comparison groups, designing longer intervention periods, and including both male and female athletes. In addition, if the underlying mechanisms of INT are to be discussed in greater depth, future research should incorporate direct physiological, neuromuscular, or biomechanical measurements to better explain the performance changes associated with INT.

## 5 Conclusion

The effects of a three-week INT intervention on the fitness and punching ability of elite male boxers were examined in this study. The results showed that short-term INT was associated with improvements in several aspects of physical fitness and punching performance. Significant improvements were observed in upper- and lower-body maximal strength, CMJ, 400 m performance, coordination and agility tests, and both single and continuous punching performance. No significant changes were observed in the 30 m sprint, 3000 m run, or rear uppercut performance. These findings suggest that INT may be a useful conditioning strategy during the preparatory phase for highly trained male boxers. However, because this study used a single-group pre–post design, the observed changes should be interpreted cautiously and should not be attributed exclusively to INT.

## Data Availability

The original contributions presented in the study are included in the article/supplementary material. Further inquiries can be directed to the corresponding author.
